# Cell and Tissue Nanomechanics: From Early Development to Carcinogenesis

**DOI:** 10.3390/biomedicines10020345

**Published:** 2022-02-01

**Authors:** Mikhail E. Shmelev, Sergei I. Titov, Andrei S. Belousov, Vladislav M. Farniev, Valeriia M. Zhmenia, Daria V. Lanskikh, Alina O. Penkova, Vadim V. Kumeiko

**Affiliations:** 1 Institute of Life Sciences and Biomedicine, Far Eastern Federal University, 690922 Vladivostok, Russia; shmelev.m.e@gmail.com (M.E.S.); titov.si@dvfu.ru (S.I.T.); belousov.ands@gmail.com (A.S.B.); frear.far@mail.ru (V.M.F.); valeri3066@gmail.com (V.M.Z.); lanskikh.dv@dvfu.ru (D.V.L.); penkova-ao@dvfu.ru (A.O.P.); 2 A.V. Zhirmunsky National Scientific Center of Marine Biology, Far Eastern Branch, Russian Academy of Sciences, 690041 Vladivostok, Russia

**Keywords:** cytoskeleton, mechanotransduction, FAC complex, YAP/TAZ, microfilaments, intermediate filaments, microtubules, cancer, carcinogenesis, stem cell niche, extracellular matrix

## Abstract

Cell and tissue nanomechanics, being inspired by progress in high-resolution physical mapping, has recently burst into biomedical research, discovering not only new characteristics of normal and diseased tissues, but also unveiling previously unknown mechanisms of pathological processes. Some parallels can be drawn between early development and carcinogenesis. Early embryogenesis, up to the blastocyst stage, requires a soft microenvironment and internal mechanical signals induced by the contractility of the cortical actomyosin cytoskeleton, stimulating quick cell divisions. During further development from the blastocyst implantation to placenta formation, decidua stiffness is increased ten-fold when compared to non-pregnant endometrium. Organogenesis is mediated by mechanosignaling inspired by intercellular junction formation with the involvement of mechanotransduction from the extracellular matrix (ECM). Carcinogenesis dramatically changes the mechanical properties of cells and their microenvironment, generally reproducing the structural properties and molecular organization of embryonic tissues, but with a higher stiffness of the ECM and higher cellular softness and fluidity. These changes are associated with the complete rearrangement of the entire tissue skeleton involving the ECM, cytoskeleton, and the nuclear scaffold, all integrated with each other in a joint network. The important changes occur in the cancer stem-cell niche responsible for tumor promotion and metastatic growth. We expect that the promising concept based on the natural selection of cancer cells fixing the most invasive phenotypes and genotypes by reciprocal regulation through ECM-mediated nanomechanical feedback loop can be exploited to create new therapeutic strategies for cancer treatment.

## 1. Introduction

Different cells formed from the same or different germ layers communicate by complex biochemical and mechanical interactions. Chemotaxis and biochemical signaling are the key mechanisms of distant interactions in various organs, tissues, and cells, and this facilitates the neurohumoral regulation of cell-tissue-organ system functionalization. This “chemical language” is suitable for communication within large groups of cells as the ligands are able to interact with the receptors located on different types of cells. Mechanosignaling is a short-range interaction between adjacent cells, or between cells and the extracellular matrix. Cells may modify their gene expression levels depending on the environmental properties, such as stiffness, Young’s modulus, and roughness [1]. It can also be triggered by blood flow [2], muscle contraction and stretching, joint flexion, and general mechanical load [3].

These local interactions have a different meaning in the context of tumorigenesis. Most of tumors start as a compact neoplasm, which is characterized by the internal interactions between its extracellular matrix (ECM) and cells, but the progression of the tumor depends on its interaction with its healthy environment [4]. Moreover, angiogenesis and vascularization are necessary for successful neoplasm growth and development. The tumor focus should be “attractive” for these processes [5], and provide optimal mechanics for vascularization by assembling the cytoskeleton and forming intercellular junctions [6]. Cytoskeletal rearrangements are widespread in tumor cells [7]; they are necessary for tumor cell migration and promote their taxis and spreading.

However, cellular mechanics does not depend only on cytoskeletal complexes, but also on membrane lipid composition, the presence of lipid rafts and their quantity, as well as on organelle ultrastructure and localization [8].

Tumor growth is vividly characterized by its uncontrolled extensive accumulation of somatic mutations in cells, and the genetic adaptations leading to an increase in tumor aggressiveness. In this review, we compare the development of a malignant tumor and the early development of an organism; in both cases, this is development starting from a single cell, from which the descendants change their phenotype depending on their microenvironment [9]. Importantly, embryonic development and tumor growth have a conceptual difference regarding differentiation or dedifferentiation.

Tumor tissue mechanics is one of the key factors in the development of the primary tumor focus and formation of metastases [10]. Due to the constant exposure of cancer cells to unfavorable factors, they are subject to the ongoing selection of cells that are best adapted to the microenvironment, and at the same time are able to modify it to ensure further proliferation [11].

In this regard, various proteins, cellular structures, and ECM components, which facilitate cancer progression and provide a favorable microenvironment, can be potential targets for therapy. Thus, it becomes important for a researcher to understand how to trace the various phenotypic characteristics of a tumor, including: the accumulation and expression levels of proteins and non-protein structures, their assembly and influence on signaling pathways, and their rheological characteristics (stiffness, elasticity, presence of pores, tendency to degradation). Mechanical mapping can be performed using various methods, both non-invasive, such as ultrasound and magnetic resonance elastography, and laboratory-based, involving biopsy and further investigations by atomic force microscopy, optical tweezers [12], and flow rheometry [13]. Nanomechanical analysis and mapping (i.e., by atomic force microscopy), as large-scale cellular analyses, are vitally important as methods for laboratory-based investigations of tumor material, which can potentially reveal patterns between the expression of certain markers and cellular rheology [14].

Despite the fact that a large number of experimental works have recently been devoted to the study of the mechanics and mechanosensitivity of cells and tissues, and several reviews provided systemic summaries, some links between important molecular components of tissue mechanics have not been emphasized enough. In this review, firstly, we give a detailed analysis of the various components of the cytoskeleton, without focusing solely on the actomyosin contractile apparatus, demonstrating its connections with the systems of microtubules and intermediate filaments that mediate cell signaling and regulation through the state of chromatin. Secondly, we describe how the cellular components and the ECM maintain the dynamic communication and mechanotransduction, also pointing out the similarities and differences between carcinogenesis and early embryonic development. Thirdly, we discuss the nanomechanical transformations occurring in carcinogenesis, with an emphasis on the natural selection of cancer cells fixing the most invasive phenotypes and genotypes through a nanomechanical, ECM-mediated reciprocal feedback loop that can be exploited to create new cancer treatment strategies.

## 2. Molecular Mechanisms and Predictors of Cell and Tissue Nanomechanics

### 2.1. Cytoskeleton Role

The cytoskeleton is a key factor that defines the nanomechanical properties of the cell surface. The cytoskeleton plays multiple roles in a cell, including the transport of intracellular structures, such as macromolecules and microvesicles; it is involved in the organization of DNA and chromosomes, cell transport, and division. Another role of the cytoskeleton is maintaining intercellular junctions, such as the tight junction, gap junction, adherent junction, and desmosomes, which are important features of animal tissues. Thus, intermediate cytoskeletal filaments provide specific types of communication between cells, which are important factors of cell–cell recognition and differentiation during ontogenesis [15,16]. The cytoskeleton plays an important role in mechanosignaling, which modifies cell metabolism, migration, and proliferation. Due to the active participation of the cytoskeleton in cell mechanosignaling processes, it has a direct influence on the regulation of gene expression by influencing nuclear lamins [17,18,19]. Cell and cytoskeletal nanomechanics contribute towards not only normal cell growth, but also to cell malignization and tumor growth. Capaci et al. (2020) [20,21] showed that in TP53-mutant tumors, the mut-p53/HIF1α/miR-30d protein cascade activates the release of proteinases, which remodel the ECM, consequently affecting mechanosignaling and promoting the metastasis process. Similar to the ECM, which may be affected by P53, the cell cytoskeleton can be restructured through the activation and regulation of integrin and cadherin pathways [22]. Normal P53 facilitates cytoskeleton remodeling to suppress tumor growth by regulating the cadherins and Rho GTPases [23]. These novel studies show that cytoskeletal organization may be a marker of cellular senescence [24] due to the upregulation of *ACTA2* gene in fibroblasts and vascular smooth muscle cells.

There are three main types of cytoskeletal molecules: intermediate filaments (IF), actin filaments, and microtubules. Together, they are responsible for the cell shape and cell membrane mechanics. Animal cells, by having the most advanced assortment of cytoskeletal molecules, may easily restructure themselves in response to environmental factors and mechanical impact. Cytoskeletal fibers can assemble and disassemble—polymerization and depolymerization of actin filaments and microtubules work as cellular “muscles” providing cell movement and migration.

Intermediate filament (IF) proteins are encoded by more than 70 genes, and most of them are keratins (approximately 54 of the 70 genes). Type I IFs are acidic keratins and type II IFs are basic keratins; these properties are defined by their amino acid sequence composition (please see the Table S1) [25]. Structural disturbances in keratins (cytokeratins) caused by various factors, both genetic and epigenetic, lead to the disruption of the formation of tissue barriers. Their expression is strongly connected to epithelial differentiation; thus, they play a large role in the processes of malignization and disease. Furthermore, some proteins of this class, such as KRT17, are able to activate cellular pathways, leading to increased proliferation [26]. Various keratins, including KRT14 and KRT15, are prognostic markers for various malignant tumors, and are used as markers for the onset of malignancy [27,28]. Moreover, these proteins have the role of maintaining cellular and tissue mechanical properties; they are proteins of intermediate filaments involved in the formation of desmosomes, the importance of which is confirmed by the fact that point mutations in these proteins are the main pathogenetic factor in the occurrence of epidermolysis bullosa [29]. The main property of keratin is its ability to form long, semi-flexible 10 nm fibers by self-dimerization, leading to the subsequent formation of the tetramer antiparallel structure [30]. These fibers form a complex supramolecular structure that provides molecular and mechanical stability to epithelial cells [31]. Epidermolysis bullosa simplex (EBS), a skin-fragility disorder, is mostly determined by the K14R125C hotspot mutation, which affects the mechanical properties of the keratin fiber structure [32,33].

Type III IFs are represented by several proteins: vimentin, glial fibrillar acidic protein (GFAP), desmin, and peripherin. GFAP is mostly expressed in brain regions, such as the cerebral cortex, cerebellum, hippocampus, and caudate, but is also expressed in the testis. It is a key protein involved in the mechanical properties of the cytoskeleton in glial cells (mostly astrocytes), allowing them to maintain the environment of the neurons to support the brain-blood barrier [34]. Its significant role in this process may be indirectly confirmed by the negative correlation of the rate of GFAP expression with the grade of tumor [35], but it may also correlate with the lower rate of differentiation and induced stemness; thus, the low expression of GFAP may be explained by cellular rejuvenation [36].

Various studies have shown that vimentin influences different cells in different ways [37]; therefore, it cannot be considered a universal nanomechanical marker, though it is strongly expressed in various cell types and organs. According to the Human Protein Atlas, vimentin is not only expressed in several types of organs and tissues, including the cerebellum (neural tissue, neuronal cells), parathyroid gland (glandular cells), oral mucosa (squamous epithelial cells), salivary gland (glandular cells), esophagus (squamous epithelial cells), gallbladder, stomach, duodenum, small intestine, and rectum (glandular cells), but also in colon endothelial cells and the peripheral nerve/ganglion, in which high or medium expression, respectively, has been detected. However, this protein is not detected in smooth muscle cells or skeletal muscle fibers. Vimentin is also widely expressed in tumors, same as in healthy tissues. According to the Tumor Cancer Genome Atlas and the Human Protein atlas, nearly all cancer groups exhibit at least some expression of its mRNAs. The protein was IHC-detected in nearly all types of tumors, excluding prostate cancer, ovarian cancer, colorectal cancer, and carcinoid cancer; in the case of renal cancer, high vimentin expression is associated with lower survival probability. The elevated expression of vimentin is actually a marker for favorable cancer prognosis.

Vimentin maintains a strong hierarchy in its composition; its properties correlate with its structural organization. Thus, unfolding alpha-helical domains may absorb mechanical energy, while keeping the other cellular structures intact. In comparison with various other mechanisms of energy transformation, vimentin can sustain 300% deformation without catastrophic failure, while inducing a continuous increase in mechanical stiffening as the tensile deformation increases. Vimentin participates in the transmission of a mechanical impulse to different parts of the cell by binding to actin, and causes compensatory contractile reactions of cytoskeletal proteins. Vimentin is also able to inflict mechanical stress on a cell due to its ability to stretch and restore back to its normal conformation [38], and on the motoric functions of muscle cells by forming Z-discs in the sarcomeres [39]. The knockout of the desmin gene results in sarcomeric ultrastructural changes and the reduction of active and passive muscle force [40]. Desmin nanomechanics corresponds to non-linear elasticity and sawtooth-like force curves, as can be observed by atomic force microscopy measurements. This means that elongation due to tension does not lead to a linear increase in stress, but is abrupt, thus changing the shape of the force curve. This can be explained by the large number of ionic interactions among desmin filaments, which may be explained by structural reorganization, which corresponds to chains rupturing and forming a new bond down the chain. There is a potential for the slippering of the desmin head domains along the coiled-coil rod of the neighboring dimer towards new binding sites [41].

IFs belonging to type IV include three types of neurofilament (NF) proteins. These are NF-L (light), NF-M (medium), and NF-H (heavy neurofilaments). They constitute the main type of IF in neurons, both mature and developing (the latter have mainly α-internexin, which is expressed in neurons at the early stages of development prior to other neurofilament proteins). NF proteins are considered to play a vital part in supporting the structure and integrity of axons, especially those of motor neurons [32].

In the peripheral nervous system, NF proteins are considered to be replaced by the peripherin belonging to the type III IFs. Recent atomic force microscopy studies on neurofilament mechanics have shown the 2.5-fold stretching ability of various purified NFs, which is very close to the properties of keratin IF and desmin [42].

**Figure 1:**
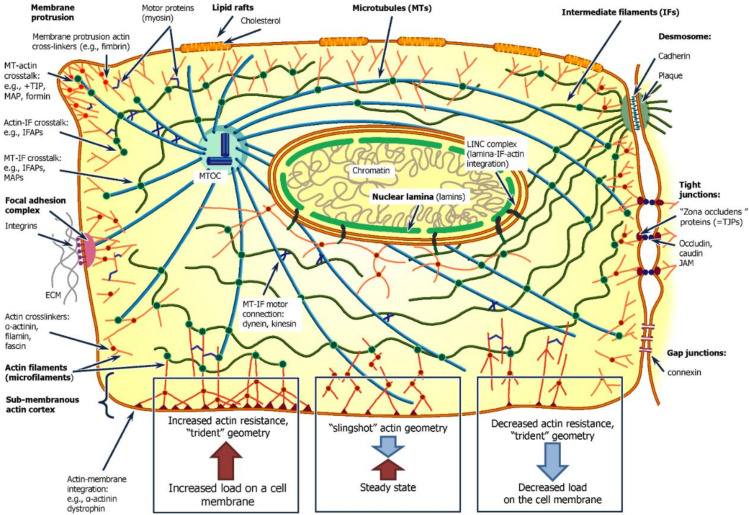
A schematic visualization of main factors influencing and maintaining cell and tissue mechanics. Cell and tissue mechanics are supported by a developed tissue skeleton system. The cytoskeleton is represented by three types of filaments—microfilament, intermediate filaments, and microtubules. These fibrillar structures are intensively interconnected with each other by various crosstalk proteins, and are also anchored to the cell membrane, facilitating intercellular contact. The cell surface system is also responsible for cell interaction with the extracellular matrix, which, by complex processes of mechanoinduction, unites extracellular and intracellular skeletal elements into an ensemble of coordinated structures. Varying force of mechanical load onto the cell surface is compensated by recruitment and reassembly of cortical cytoskeleton. The cytoskeleton also serves as a mediator, helping to sense extracellular mechanical signals and transduce them to initiate genetic response.

The type V IFs are nuclear lamins, forming a special cytoskeletal entity—nuclear lamina (see Figure 1). They are specific proteins that play a large role in chromatin organization. They are divided into two subtypes—the A family (including lamins A and C, which are splice variants of a single *LMNA* gene) and the B family (Lamin B1 and Lamin B2, encoded by *LMNB1* and *LMNB2* genes). Purified lamins have the ability to form supramolecular higher-order filamentous structures in vitro [43], which involves a multi-step process started by dimerization and head-to-tail interactions of coiled-coil dimers to form protofilaments [44,45].

The VI class of IFs includes only one type, nestin, expressed during the early development of neurons in stem cells of the central nervous system. Recent studies have shown that nestin knockdown in a cell culture in vitro decreases the migration ability of neural stem cells by reducing cellular contractility [46].

Actin fibers, or microfilaments (F-actin), are polymer fibrillar structures composed of globular actin molecules (G-actin). These proteins have a peculiar property of self-assembling and self-disassembling through the activation of various actin-binding proteins. In mammals (including humans), actin is represented by six types, encoded by six genes: *ACTA1, ACTA2, ACTB, ACTC1, ACTG1, ACTG2*. These isoforms have a very similar nucleic acid sequence, possessing a 92% similarity with the least similar part near the N-terminal. However, these genes are expressed differently in various organs (please see the table in the Supplementary Materials), which may indirectly prove their various roles in providing cellular motility and muscular contractility. According to the functions of these proteins, actin types are classified as cytoplasmic (β- and γ-cytoplasmic actin) and skeletal (α-skeletal, α-cardiac, α-smooth, and γ-smooth actin). The sequence identity of these groups is also higher than in the rest of the actin family: 98% and 97%, respectively [47].

The actomyosin complexes may drive membrane motility and affect the membrane proteins [48]. The ultrastructural organization of actin fibers is also important for the stability and mechanical rigidity of any particular cell. T. Svitkina (2019) [49] previously reviewed various predicted and observed structural forms in actin networks, which depend on the mechanical interaction between plasma membrane and its environment. Thus, the “slingshot” geometry, with an angle of 35° between two main actin filaments interacting with the plasma membrane, which is usual for an intermediate rate of mechanical interaction, may be converted to a trident geometry with higher filament density and more branched ends. Thus, a larger degree of interaction between actin filaments and the plasma membrane can be achieved in the case of tighter interactions. It can also be converted to a trident-like actin network with a lower density and predominant ~0° angle (see Figure 1). Such a change in the geometry of the actin filaments may indicate a compensatory reaction of the cells to a mechanical stimulus; additionally, the stretching or compression of the cell membrane causes tension in the proteins of the cytoskeleton and can cause them to stretch, which downregulates the Hippo pathway and leads to the release of the YAP/TAZ complex from the nucleus [50] and the inhibition of cell proliferation.

Another class of cytoskeleton fibers microtubules also has a significant role in cellular mechanics and the maintenance of cell structure. These fibers are composed of αβ-tubulin, and may dynamically assemble by the addition of a tubulin monomer conjugated with a GTP molecule [51]. The growing microtubule generates a directional mechanical force, which can push and move various large cellular structures, for example nuclei, to a growing terminal, or pull them from a shrinking cell terminal [52]. However, these microtubes are highly stiff—their mechanical properties are close to those of the F-actin backbone and have a high curvature rigidity due to their large diameter. In response to mechanical impact, tubulin fibers organize into short-periodic bends, with lengths of 3 µm. This is possible due to the surrounding and coupling of these fibers with other matrix fibers (see Figure 1) [53], such as the AtFH14 interactions between actin filaments and microtubules [54].

Cytoskeleton fibers play a major role in ultrastructural organization of the cellular cytoplasm and nucleoplasm, and form a specific structure that involves all four biological compounds: lipids, proteins, carbohydrates, and nucleic acids [55,56].

### 2.2. Lipid Membrane Organization

The membrane lipid bilayer is very flexible and fluid, and so it has little effect on the mechanical properties of the cell membrane. However, some studies show that there are several factors that can stabilize its structure and increase its rigidity, for example: changing the content of cholesterol in the lipid bilayer [57]; modifying its shape by curving membrane structures by scaffolding, introducing hydrophobic insertions, oligomerization, and steric effects [58]; utilizing lipid rafts capable of serving as reservoirs of high melting lipids, and that act as thermal buffers of membrane physical properties [8]. On the other hand, there is a large influence of the lipid membrane organization on membrane flexibility and fluidity. The influence of hydrophobic molecules, and molecules with low transition temperatures, drastically affects the motility of the cells in a case of lower environment temperatures [59].

### 2.3. Nuclear Organization

The nucleus, an organelle with its own cytoskeletal system mostly represented by the type V IFs, differs significantly from all other cytoplasmic structures. Its cytoskeleton is specifically connected to cytoplasmic cytoskeletal structures and also to histones. This fact may prove the theory that the cell niche, including the neighboring cells and the ECM, may influence the cell cycle and induce certain genes [60,61].

Nuclear mechanical properties are based on the properties of its envelope and lamina. These structures determine the mechanical properties of the nucleus; its Young’s modulus ranges from 0.1 to 10 kPa, and it may be defined by the different composition of the lamina protein structure and the “specialization” of different lamina protein types. Thus, A-type lamins control the viscosity of the lamina, allowing the nucleus to maintain its structure under the influence of the force applied, and B-type lamins repair the nucleus and restore its structure [62]. Therefore, nuclear composition may modulate the leukocytes’ ability for migration and infiltration into the nearby structures. Lamins, as a part of the nuclear matrix, are necessary for direct chromatin rearrangement, with the participation of the LINC (Linker of Nucleoskeleton and Cytoskeleton) complex, and they can also be a factor facilitating cell migration through epithelial barriers by reducing the rigidity of the nucleus, as demonstrated for leukocytes [53]. It was also found that, at high levels, lamin B1 can promote cell migration by altering perinuclear actin organization [63], and should be further investigated as a potential factor inhibiting cancer cell migration and metastasis [64]. Moreover, the structure of the nucleus may be a limiting factor for cell migration [65].

The destination of cell migration may be predicted by cell morphology and polarity. In wound-healing assays, conducted in vitro on a flat surface, the nucleus is usually placed on the rear terminus of the cell, while the other organelles are located on the leading end [66]. If it is impossible to determine cell polarity, the migration processes are also difficult to identify. Due to actin fibers serving as the main fulcrum for cell motility, it is important to provide steady junctions between lamina structures and microfilaments, to provide the linker of nucleoskeleton and cytoskeleton complex [67]. T. J. Chancellor et al. (2010) showed that HUVEC cells lost their wound-healing ability [68] via the siRNA knockdown of nesprin-1, one of the proteins that connects the nucleus to the actin filaments [69].

Similar to how lamina influences the mechanical properties of the nucleus and the whole cell; the chromatin architecture may also determine cellular mechanics. Depending on the shape and type of DNA 3D structures—the rate and the type of compactization—the DNA molecule may absorb the mechanical energy, which pulls the fiber and unfolds it without the breakdown of the chain [70]. The chromatin structure also includes specific linker DNA that connects the nucleosomes and potentially play a major role in DNA assembly [71], and may also absorb various mechanical torsion impacts [72]. Structural chromatin proteins condense DNA molecules and stabilize its shape and structure [72]; thus, the enriched regions may have other nuclear mechanics, and the mechanical stress may rearrange the DNA and change its transcription profile [73]. It has also been shown that heterochromatin is able to protect the genome from stress by softening the nucleus due to the reorganization of the constitutive nanoarchitectonics and nanomechanics of cellular chromatin, and, as a result, reducing the tension of the nuclear envelope [74].

## 3. Cell Mechanoreception and Behavior

### 3.1. Cell Mechanoreception and Responding to Mechanical Signals

Cells can convert mechanical signals into biochemical signals using mechanotransduction systems [75]. This possibility is due to interactions between integrins and several adapter proteins, which form focal adhesion complex (FAC), including focal adhesion kinase (FAK), p130Cas, paxillin, talin, and vinculin.

**Figure 2:**
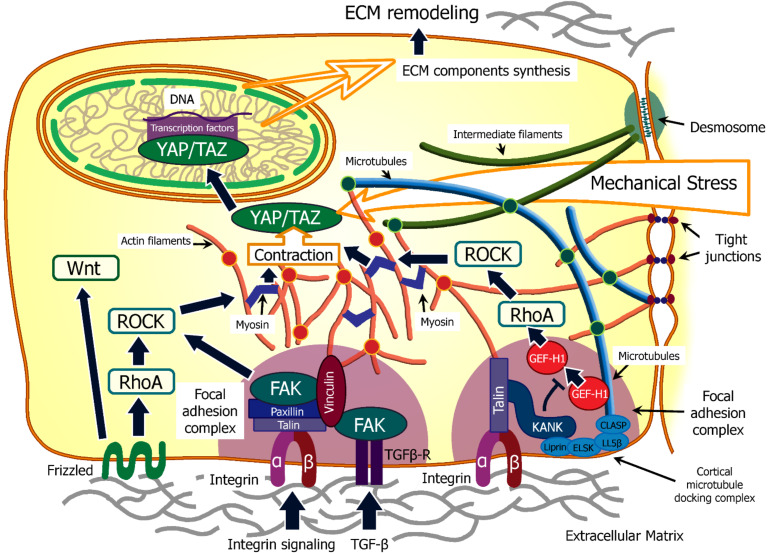
A schematic visualization of molecular pathways activated by mechanoreception. A cell possesses a developed system of mechanical receptors, including complexes contacting the ECM (extracellular matrix) and cell junctions. The majority of these receptors induce the ROCK-based signal transduction mechanism, which initiates actomyosin contraction. This mechanochemical response induces activation and transfer of the YAP/TAZ complex to the cell nucleus to influence gene expression. This may lead to synthesis of various components of both cytoskeleton and the ECM, therefore stimulating the ECM remodeling and reconstruction, therefore launching the complex feedback loop of tissue skeleton dynamics. This mechanism can be induced by a direct mechanical impact on any components of the cytoskeleton that are integrated into the membrane-associated complexes, and also interconnected with each other in the cytoplasm.

These proteins are part of the focal adhesion complex, which, in addition to its receptor function, is directly associated with actin filaments. The activation of these complex leads to the activation of Hippo pathways, and as a consequence, a cellular response to mechanical action [76]. Cells determine the stiffness of the surrounding extracellular matrix using the p190RhoGAP protein, which inhibits RhoA GTPase, an enzyme that promotes the formation of membrane protrusions and an increase in cell motility. A decrease in the number of p190RhoGAP leads to a change in cell morphology and a more rounded shape, which, in combination with a decrease in the number of integrins, can reduce the contact of cells with the ECM (Figure 2) [77].

Focal adhesion contacts are special complexes that connect the ECM with the cytoskeleton. Thus, hyaluronic acid, laminin, or fibronectin of the ECM interact with the integrin receptors. This interaction triggers various signal transduction pathways to promote cell survival, growth, and division by recruiting various kinases, such as FAK [65].

Integrin receptors can also be connected to the contact points of the cortical microtubules with the cell membrane, through the KANK protein. In the absence of mechanical stress, this protein inhibits the release of the GEF-H1 factor, which, when released, is able to trigger Rho/ROCK signaling and lead to the activation of the Hippo pathway, through a mechanism similar to the activation of the FAK-dependent pathway (Figure 2) [78].

Like other controls for cell division, control of attachment to solid substrates works in the G1. Cells need to transit from the G1 to the S phase, but anchoring is not required to complete the cycle. In fact, cells usually decrease their adhesion to the substrate and round out as they pass through the M-phase. This attachment and detachment cycle allows cells in the tissues to re-arrange their contacts with other cells and with the extracellular matrix. In this way, daughter cells form contacts with the surrounding tissue before they are allowed to begin the next cycle of division.

There are two-way interactions between the extracellular matrix and cells. On the one hand, cells themselves synthesize the extracellular matrix and participate in its organization. The geometrical arrangement and orientation of collagen depends on the direction of migration of the cells that synthesize collagen [79]. On the other hand, cells constantly perceive a whole spectrum of signals that come not only from other cells in the body, but are also provided by the microenvironment. These signals include, for example, chemical composition, topographic and viscoelastic properties of ECM, and the molecular structure of adhesive ligands. The reactions of the cells to these signals can be a change in the shape of the cell, its polarity, and its metabolism. For example, the polarization of fibroblasts during growth in culture depends on the stiffness of the substrate [80]. Substrate stiffness can initiate the synthesis of other ECM components indirectly, by activating a growth factor, or directly, by triggering an intracellular signaling pathway that activates the transcription of structural genes [81].

Thus, due to the mutual ordering of the cytoskeleton of cells and matrix macromolecules, the ECM plays a central role not only in the creation and maintenance of the structure of tissues and organs, which is especially important in the processes of development and regeneration, but also regulates tissue morphogenesis through the selective activation of gene transcription.

The cell–ECM interaction can be carried out through focal adhesion contacts connecting the ECM with actin filaments, podosomes, or through semi-desmosomes and fibrillar adhesions, in which the ECM components are connected with intermediate filaments (Figure 2) [82].

### 3.2. The Role of ECM Physical Properties on Cell Functions

The nanomechanical properties of the extracellular matrix are determined by various parameters, such as stiffness, modulus of elasticity, the presence and size of pores, and a tendency for degradation. All these characteristics can be explained by the different composition of the cell matrix, the inclusion of large and small molecules, and the covalent and non-covalent interactions between them [76]. Covalent bonds provide high rigidity, a linear dependence of elasticity on stretching, and low mobility; however, weak bonds will affect the fluidity of the membrane, being easier to break yet more numerous and more prone to rearrangement. Depending on their molecular weight and concentration in the matrix, small molecules can lead to energy dissipation and a decrease in the ability of the matrix to restore itself after mechanical stress [83]. These characteristics have found practical implementation in the design of synthetic gels, e.g., pectin-based hydrogels [21] or modified polyethylene glycol (PEG) [84]. Both these types of gels are applicable in the process of rehabilitation after an injury or surgery. Combinations of different monomers at different concentrations can be used to achieve different mechanical properties, making them potentially applicable to the replacement of matrices of various tissues, and leading to an increase or slowdown in proliferation, as well as differentiation. Polyurethane gels have also been shown to self-heal after loading, presumably by repairing disrupted ionic bonds [85].

**Figure 3:**
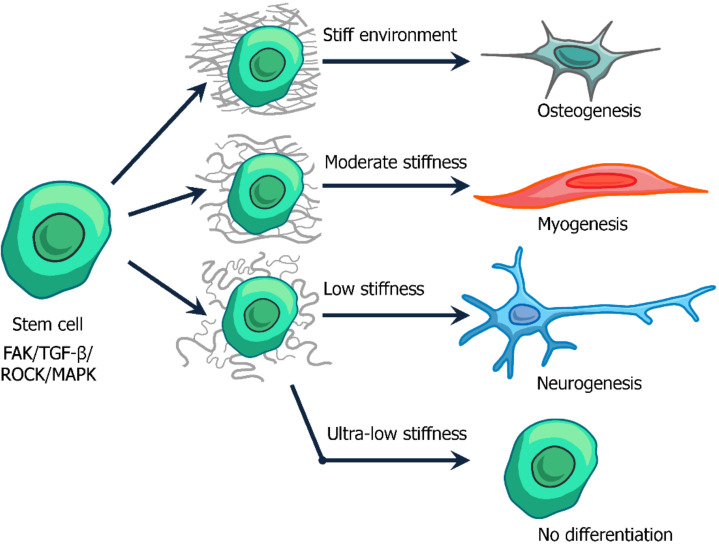
Possible pathways of mesenchymal stem cell differentiation in response to mechanical properties of extracellular matrix. The softest materials not only support the cellular stemness, but inhibit cell division processes.

ECM stiffness has a direct impact on the differentiation of stem cells of various cell types [86] (see Figure 3).

Cells cultured in Matrigel tended to differentiate to the glial pathway when the Young’s modulus ranged from 1 to 10 kPa, and to neurons with the Young’s modulus ranging from 0.1 to 0.5 kPa. With Matrigel’s Young’s modulus as low as 0.01 kPa, neural stem cells could no longer proliferate or differentiate [87].

The impact of the ECM Young’s modulus is also important for naive mesenchymal stem cells, which can undergo neurogenesis, myogenesis, or osteogenesis under conditions of 1, 10, or 100 kPa, respectively. These are Young’s modulus levels typical for brain tissues, muscle, and bones, respectively (see Figure 3) [71]. Differentiation of skeletal muscle requires cell–ECM interactions [88]. Muscle stem cells, which are naturally found in adult tissues, proliferate rapidly in vivo, but do not proliferate as quickly when cultured on rigid plastic dishes that have a Young’s modulus of 106 kPa. However, if cells are cultured in 12 kPa Matrigel that mimics muscle stiffness, the muscle stem cells will continue to self-renew in vitro. In addition, these cells can be transplanted into mice and greatly aid in muscle regeneration [89].

ECM stiffness regulates chondrocyte differentiation through ROCK signaling and TGF-β activation. Chondrocyte differentiation requires Smad3 phosphorylation, which is optimized at the same ECM rigidity as induction of chondrocyte gene expression [90]. In all these cases, the interaction occurs through adhesion complexes, which are based on integrins—the main receptors for the interaction of cells with ECM (Figure 2 and Figure 3).

Integrins perform not only cell adhesion, but also signal transduction. On the one hand, the interaction of molecules of the extracellular matrix with integrin triggers responses in the cell. On the other hand, the cell can regulate the degree of binding of integrins to ECM molecules through internal signals [91]. Integrins directly activate survival pathways via the PI 3-kinase and MAPK pathways, and act as important cofactors for their stimulation by growth factors. Moreover, increased expression of integrin in the absence of suitable ligands, or in the presence of natural or synthetic antagonists, can promote apoptosis under other conditions allowing growth [92].

It is assumed that the simultaneous interaction of multiple integrin receptors with the binding sites of the extracellular matrix allows cells to collect topological descriptions of the chemical and mechanical properties of their environment. This information is then converted into intracellular signals that affect the position, differentiation, and growth of cells, as well as other fundamental processes, such as protein synthesis and the regulation of energy metabolism [93].

In addition to integrins, non-integrin receptors facilitate the cell’s connection with the extracellular matrix. The best studied of these are syndecans, which are fixed in the membrane by the core protein. The extracellular domain has three to five heparan sulfate or chondroitin sulfate chains, and the intracellular domain is associated with the actin cytoskeleton. Syndecans bind ligands, such as fibroblast growth factor, preventing degradation, and present them to specific receptors on the cell surface [94].

The composition and physical properties of the ECM are not the only factors that can lead to the transmission of information by means of mechanotransduction. The nanotopography (supramolecular structure) of the ECM is able to influence differentiation, migration, and proliferation [95]. The supramolecular structure of the ECM changes the organization of the cytoskeleton of the vascular endothelium [96] and human mesenchymal stem cells [97]. This occurs via the FAK signaling pathway [98].

## 4. Nanomechanical-Based Differentiation and Specification of the Cells during Ontogenesis

### 4.1. Blastocyst

The mechanical forces that affect the formation of the fetus in the early stages of embryonic development include two main factors that affect the behavior of cells: the mechanical rigidity of the local tissue environment, including the cells and the extracellular matrix, and the contractile activity of the cells of the microenvironment. These properties contribute to the occurrence of mechanical stresses in cells, which are necessary for mechanotransduction. (see Figure 2) [99].

Rigidity plays an important role during embryogenesis. It was found that culturing embryos on a softer surface (collagen) led to the development of two-cell embryos up to a blastocyst, while cultivation on softer materials increased the frequency of hatching and the number of trophoectoderm cells compared to embryos cultured on a harder surface (polystyrene). It was confirmed that fetuses obtained from embryos grown on collagen in vitro had a greater placental weight at E12.5 (“E” is the fetal to placenta (F:P) weight ratio at embryonic day) [100].

**Figure 4:**
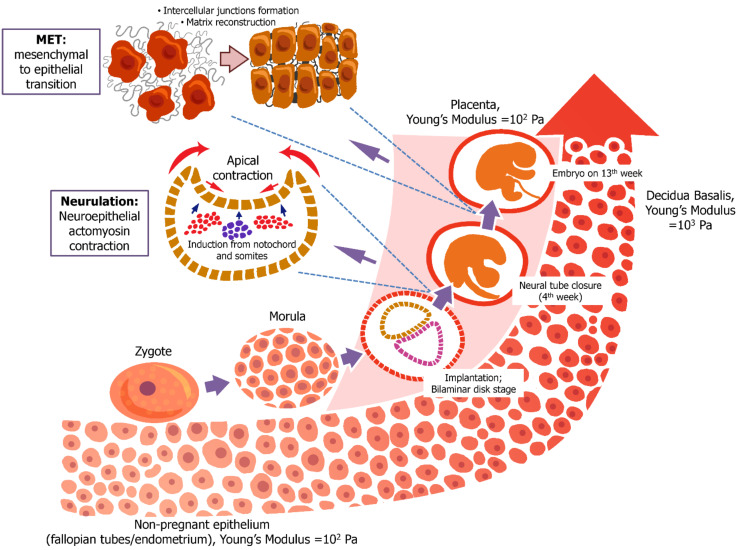
Embryonic mechanosensing promoted by mechanical parameters of tissue microenvironment. Early embryogenesis requires soft environment and intercellular mechanosignaling promoted by contraction of actomyosin cortical cytoskeleton. Further development is characterized by increased stiffness of decidua. However, cellular contractility and formation of intercellular junctions are still important for cellular differentiation.

However, further embryonic development requires a stiffer substrate. For example, Abbas et al. measured the stiffness of ex vivo samples of the secretory endometrium of a non-pregnant person, the basal decidual membrane of the first trimester, and the decidual membrane of the parietal and placenta. It was found that the stiffness of the basal decidual membrane examined in the first trimester was 10^3^ Pa, while the stiffness of the non-pregnant endometrium, parietal decidual membrane, and placenta was ~10^2^ Pa. It was suggested that a stiffer substrate is required for a higher rate of proliferation, and may be needed for appropriate differentiation (Figure 4) [101].

Cell proliferation may also be regulated by mechanical stresses provided by cellular actomyosin contractility, which can be transmitted through the tissue. It is important to note that the mechanical impact influences embryogenesis starting from the earliest stages of blastocyst formation. Although cell division is necessary for the formation of a blastocyst from a zygote, the shortened cell cycle that characterizes proliferation at this stage is regulated independently of intercellular interactions. Thus, there is an assumption that cytoskeletal tension is a strong regulator of proliferation [99].

It is also worth noting the influence of the volumetric and mechanical characteristics of the blastocoel. They can affect the process of division of trophectoderm cells and thereby affect the cellular distribution and fate. It has been shown how blastocoel-mediated, mechanical cell-to-cell interaction and tissue rheology control the size of the embryo at the tissue level, which is related to the position and fate of cells at the cellular level [102].

### 4.2. Primary Organogenesis and Tissue Genesis

Neurulation is an important part of primary chordate organogenesis, in which the neural tube forms and separates from the surface epiderm. Neurulation is in fact defined not only by cellular processes, but also by biomechanical mechanisms; these include, the formation and the bending of the neural tube, the lifting of nerve folds, and the fusion of the neural plate [103].

It was revealed that mechanical forces contribute to both the closure and opening of the neural tube. The disruption of dynamic morphological changes in neurulation leads to disturbances in the closure of the neural tube and subsequent severe defects in the neural system [104].

Neural tube closure depends on the myosin II sequential contraction and the functional exchange of cells along the neuro-epidermal border [105]. The migration of a deep layer of cells into the neural plate is necessary for the final closure of the neural tube after the convergence of the nerve folds [106].

The Wnt/PCP pathway is also important for neural tube closure, due to the regulation of actomyosin-dependent contractility and coordinated cell movements [103,107]. Planar cell polarity proteins (PCP) control the direction of cell polarization. Kolahi et al. (2012) [108] showed that the asymmetric enrichment of PCP proteins strongly correlates with the actomyosin-driven contractile behavior of cell–cell junctions. Furthermore, a complex and close relationship was revealed between the dynamic localization of main PCP proteins, the assembly of actomyosin, and the reduction of the polarized compound during the closure of the neural tube in vertebrates [108].

In addition to the Wnt/PCP pathways, the Grainyhead-like2 (*Grhl2*) transcription factor of the superficial ectoderm also plays a critical role in regulating the biomechanical properties of the superficial ectoderm, which is important for neurulation. The knockout of *Grhl2* triggers the change from epithelial to neuroepithelial cell identity (due to the high content of N-cadherin in the surface layer of the ectoderm). It also leads to the disorganization of actomyosin, which is responsible for the closure point of the posterior neuropore [109,110].

### 4.3. Secondary Organogenesis

In the process of organogenesis, intercellular junctions and mechanical forces in tissues can have a significant impact on the development of the embryo. Thus, cadherins, transmembrane glycoproteins of the cell surface, are involved in the mechanical connection of cells with each other, and are also important for the development of the body [111]. When compared with humans, in *Xenopus spp.*, mechanical tension forces affect the C-cadherin of the mesoderm cells, which leads to a protrusion of polarized cells; alternatively, in zebrafish, the segregation of the germ layer precursors occurs when exposed to E-cadherin. [112,113,114].

In the study of Greene, N.D.E. and Copp, A.J. on the influence of the concentration of fibrinogen or thrombin on substrate stiffness, it was found that these parameters are in a direct dependency [104]. Furthermore, a substrate with low rigidity, and with a low thrombin content, contributed to an increase in the proliferation and differentiation of endoderm cells when compared to more rigid substrates [115]. Another study [116] illustrated the mechanical role of the endoderm during the formation of the cardiac tube, as follows: the endoderm is an inductive substrate, secreting various growth factors that induce cardiac specification and differentiation; the endoderm actively contracts around the anterior intestinal portal (AIP), ensuring the correct formation of the cardiac tube.

It is worth noting the mechanical impact of the mesenchymal—epithelial transition (MET) on the morphogenesis of the epithelium. The cells participating in MET exert a mechanical effect that converts them from irregular-shaped cells to regular epithelial cells, such as tubule epithelia and myocardium epithelia (Figure 4) [117].

It was found that the ectoderm-to-mesoderm transition depends on the inhibition of ROCK signaling and the overexpression of Rnd1 and Shirin; these factors promote cell migration or mesodermal transition due to the downregulation of actomyosin contractility, which leads to the activation of the Hippo signaling pathway [118].

In addition to all the above, certain mechanical forces are involved in controlling the formation of a pattern of neuroectoderm cells. Xue et al. [119] showed that the shape of cells and the contractility of the cytoskeleton provide feedback for the mediation of bone morphogenetic proteins (BMP). Thus, spatial regulation occurs in the formation of the neuroectoderm pattern [119].

## 5. Nanomechanical Features That Provide Cancer Aggression and Invasion

### 5.1. Cellular Component

**Figure 5:**
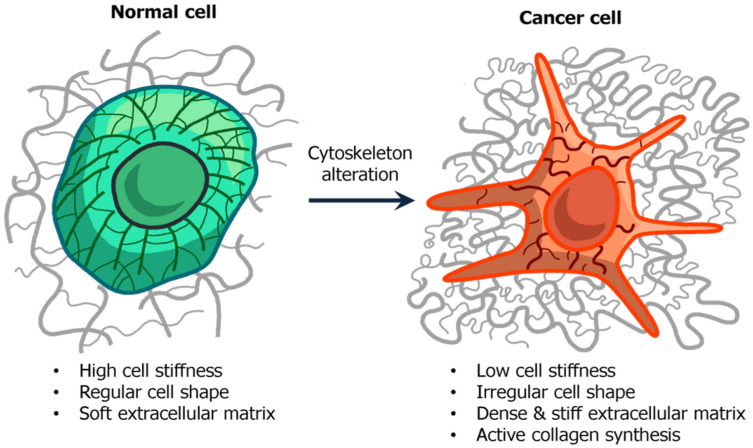
Reaggregation and reassembling of the cell—ECM (extracellular matrix) complex during malignization. The normal cells are regular-shaped and form around themselves a specifically organized structure. The cancer ECM is less organized, and may be partially disrupted due to metalloproteinases, synthesized by cancer cells.

Due to the fact that cancer cells retain their ability to proliferate over a long period of time, and are also exposed to immunity and negative factors associated with tumor growth, there is a steady generational change, and a form of “natural selection” for the most adapted clones [120]. Moreover, one can now also consider the direction of such selection; there are studies that describe the order of occurrence of mutations, which determine the survival of cancer cells and tumor progression [121]. Moreover, treatment intensifies this selection; it is known that the partial resection of a tumor, as well as incomplete therapy, promotes the selection of more aggressive cells and an increase in the degree of tumor aggressiveness—otherwise, the patient typically recovers [122]. The nanoarchitectonics and nanomechanics of cellular structures will change in such a way as to provide the greatest invasiveness, migratory, and proliferative activity. Their microenvironment should support high proliferative activity, its rigidity should be increased, but, at the same time, it should be easy to decompose, not to impede, cellular migration. However, these changes are the result of a rather long process, and require progressive tumor growth and multiple generational changes [123]. As a result, it can be assumed that a sharp change in cell growth conditions can cause cellular stress, which can lead to a decrease in growth rates, or cell death (see Figure 5) [124].

In the process of carcinogenesis, the cell cytoskeleton, which is the main effector of cell nanomechanics, undergoes significant changes, mainly concerning structure rearrangements, which leads to an irregular-shaped cell. This processes mainly occurs under the influence of disturbances in the process of differentiation and repeated, unequal cell division [125]. Therefore, in addition to the transformation of other cell organelles, carcinogenesis signifies a special mechanical phenotype of cancer cells [126].

It seems that there is a mechanism for changing the nanomechanical parameters of tumor cell adhesion. Before entering the bloodstream, cells are characterized by a decrease in the quantity of adhesion molecules, and therefore their low adhesion [127]. Activated metastatic tumor cells have two main nanomechanical features: softness and high adhesion. This allows them to effectively spread throughout the body, even surpassing biological barriers and attaching to tissues. These mechanisms are specifically activated when cells enter the bloodstream [128]. Furthermore, the greater the invasive potential of the cells, the higher their adhesion and softness [129].

When compared to normal cells, invasive tumor cells are characterized by a wide range of stiffness values due to their ability to adjust their nanomechanics under certain microenvironmental conditions. This has been proven for many types of oncological diseases [130,131,132,133].

The actin cytoskeleton plays a significant role in the adaptation of the rheological properties of tumor cells to environmental conditions. It is its rearrangement that is the key factor involved in increasing the stiffness of cells following an increase in the stiffness of the substrate. Perhaps this mechanism underlies extravasation (see Figure 5) [134].

The epithelial-to-mesenchymal transition plays a great role in a large number of cancer types, and is carried out under the influence of changes in cell nanomechanics. The expression of the vimentin and fibronectin genes increases, and cell motility increases due to the reorganization of the cytoskeleton, which leads to a significant transformation of cell mechanics [135].

It was shown that the nanomechanics of tumor cells depends on the rigidity of the substrate. Metastatic breast cancer cells are able to adapt to the stiffness of the matrix, increasing their elasticity, which allows them to survive in tissues with varying rigidity [136]. A similar change in cell stiffness has also been demonstrated in bladder tumor cells [134].

On the other hand, it was shown that the most aggressive tumor cells also become stiffer on soft substrates. The stiffness of aggressive cells becomes more pronounced when they come into contact with each other, while the stiffness of normal and less aggressive tumor cells does not change under similar conditions [137].

### 5.2. Extracellular Component

The viscoelastic properties of tissues are the result of various interactions between cells and molecules of the extracellular matrix. On the one hand, cells, during their life cycle synthesize various components of the matrix, and on the other hand, the enzymes that decompose them. Thus, cells construct the matrix with parameters matching their current needs. The mechanical signals that a cell receives when interacting with the environment regulate its behavioral processes, such as proliferation, differentiation, migration, and adhesion, through mechanotransduction [138,139].

The extracellular matrix of tumor tissues is subject to significant rearrangements (see Figure 5); for instance, there are changes in the expression levels of matrix components and in the rate of their degradation, and changes in the spatial orientation of individual molecules and their various modifications. This remodeling of the ECM results in changes in the bioavailability, activity of its signals, and its feedback to cells [139,140]. Matrix stiffness plays an important role in cancer, regulating the development of tumor transformation, the proliferation and invasive potential of the cells and their survival, as well as the formation of metastases [140].

It is known that tumor formation is accompanied by an increase in ECM stiffness, caused by the recruitment and activation of cancer-associated fibroblasts. Moreover, it was found that there is a relationship between the increased stiffness of the ECM and the metastasis process [141]. Changes observed in the ECM during tumor progression can be described as fibrosis; fibrillar collagen type I and III are the key matrix components influencing its stiffness [142]. Type I collagen is the main component of the tumor stroma, and its levels correlate with the survival of transformed cells and the development of metastases. Fibrillar collagens are more often oriented isotropically in healthy tissues, but they are anisotropically aligned in tumors [143]. Additionally, several types of proteoglycans are involved in changing the organization and mutual arrangement of collagen fibrils [139].

Furthermore, the stiffness of the matrix increases with an increase in the number of crosslinks between its proteins, such as fibronectin and type I collagen [144]. ECM proteins are more frequently crosslinked by LOX-enzymes, especially LOX-1, LOXL-2, and transglutaminase-2, which are upregulated in neoplastic tumor tissue [145].

Increased accumulation of collagen type I, III, and IV, and a large number of collagen cross-links, promote tumor development by activating integrin signaling [146].

An increase in the accumulation of fibrillar collagen and a large number of cross-links leads to the formation of desmoplasia, a stiff, fibrous deposition of ECM, characteristic of transformed tissue. Other components of the tumor stroma are tenascin-W, laminin-332, and a fibronectin splicing variant [147].

Fibronectin is often found in the extracellular matrix of tumors, especially its splicing variants, such as ED-A and ED-B [148]. Fibronectin fibers have a specific orientation in the tumor tissue. They are directed outward from the center of the tumor, and this probably potentiates invasive tumor growth. In addition, signaling triggered by fibronectin can activate focal adhesion kinase (FAK), which then activates the invasion cascade in the cell and stimulates cell proliferation [149].

Laminin-332 (encoded by *LAMA3*, *LAMB3*, and *LAMC2* genes) is also found in some types of tumors, although it is normally found in the bone marrow, which suggests its role as a molecule that promotes cell division [150].

An increased content of the carbohydrate component—hyaluronic acid—is typical for many types of tumors, including; carcinomas, brain tumors, pancreatic-, and breast cancer. It can act as a ligand for CD44, which mediates tumor invasiveness and metastasis [151].

There is evidence that matrix stiffness allows the differentiation of both benign and malignant neoplasms and precancerous conditions [142,152].

The second important property of the extracellular matrix of tumor tissue is its degradation due to the high activity of metalloproteinases, which are not very active in normal tissues [153]. Metalloproteinases destroy ECM components and allow tumor cells to actively migrate through the stiff matrix. The degradation of the matrix leads to the release of signaling molecules and growth factors that have been deposited in it, and clears their binding sites that activate cell proliferation through integrin receptors [146,154]. Metalloproteinases can exhibit anti-apoptotic activity associated with the cleavage of the Fas ligand and of MHC (major histocompatibility complex) class I, and activate angiogenesis, promoting the release of VEGF [154]. Type 2 metalloproteinase directly affects tumor cell survival by activating the STAT3 survival pathway [155].

The nanomechanical properties of healthy and tumor ECMs are already being used to create promising artificial matrices for targeted therapy in various types of cancer, and for treatment and rehabilitation after traumatic and ischemic damage of tissues, such as cartilage [156] and bones [157]. As another example, polygalacturanide matrices were developed for the restoration of nerve tissues [21]. Additionally, diagnostic approaches are being developed for effective diagnostical methods, including intraoperative methods [158]. At the same time, diagnostic evaluation of mechanical properties, such as rigidity, elasticity of organs or tissues and their mapping, is not new. Methods of macro-determination of mechanical properties by ultrasonic [159] or magnetic resonance elastography [160] have been known for quite some time. However, these methods currently cannot be used for nanomechanical mapping, although tools based on atomic force microscopy, optical tweezers, or similar technologies can be used at the level of individual cells and extracellular structures [161].

### 5.3. Tumor Stem Cells Niche ECM and Its Effect on Tumor Progression

It is not the tumor cells themselves that contribute most to the development and progression of a tumor, but rather its extracellular matrix and the niche of the tumor stem cells that it forms [155]. However, cancer stem cells play a key role in tumor initiation and maintenance [162]. Tumor stem cells in the body are located in the unique microenvironment of the tumor niche, which consists of ordinary tumor cells, non-tumor stromal cells, resident and infiltrating immune cells, other types of glial cells, and a well-developed vascular network. These cells actively synthesize various components of the extracellular matrix. Typically, many components of the brain extracellular matrix are overexpressed in tumor cells, which makes the tumor extracellular matrix denser and more structured than the normal brain extracellular matrix. The mechanical properties of the ECM affect tumor stem cells through the formation of focal adhesions and the subsequent activation of mechanotransduction pathways (e.g., Rho/ROCK, YAP/TAZ). ECM rigidity plays a critical role in regulating stem cell self-renewal and differentiation. Inhibiting YAP/TAZ- TEAD is an attractive and viable option for novel cancer therapy [163].

Tenascin-C glycoprotein, a typical component of the central nervous system development, exhibits high levels of expression in tumors; these levels are thought to directly correlate with the grade of glioblastoma multiforme (GBM) malignancy and patient prognosis [164]. In addition, glioma expresses ECM components not found in the adult brain, such as the membrane-anchored proteoglycan syndecan-2, one of the members of the heparan sulfate proteoglycan family [165]. Thus, the tumor ECM is a special type of matrix, similar in structure to the ECM of the developing brain, both in the components present and in the ability to promote cell migration, which distinguishes it from the ECM of the adult brain, where migration is strictly limited.

The physical properties of the tumor ECM, such as stiffness, porosity, and topography, affect the cancer stem cells (CSCs) interacting with it. The tumor ECM provides a physical barrier that prevents the transport of solutes, water, and most chemotherapy drugs. In this regard, it has been shown that cisplatin, a chemotherapeutic drug often used to treat various solid tumors, actively binds to fibrillar extracellular matrix proteins in tumors [166]. The binding of drugs to the ECM prevents drugs from entering tumors, thereby increasing the survival rate of CSCs.

The change in the ECM can provide external signals that lead to an epithelial-mesenchymal transition (EMT), which leads to a loss of apical–basal cell polarity, deprivation of tight intercellular junctions, and reorganization of the cytoskeleton (see Figure 5). The result is a transformation of the phenotype of cells and, most importantly, the emergence of their mobility. It is this mechanism that probably ensures the change of the subtype of GBM from proneural to the more aggressive mesenchymal. EMT is a strategically important mechanism for generating new types of CSCs and enhancing their invasive properties.

## 6. Conclusions and Future Perspectives

Cell-to-cell communication is based not only on a chemical, but also on a mechanical “language”. This is mediated and amplified by the extracellular matrix, constituting an entire tissue skeleton, where external mechanical signals can trigger gene regulation though the cytoskeleton feedback loop. Since it is the physical properties of cells and their microenvironment that actually guide the processes of normal cell differentiation and pathological pathways, this is of significant interest for both developmental biology and cancer research, and this data could be implemented in clinical diagnostics and therapy.

Cell behavior and tissue promotion are complex phenomena that contribute towards understanding the processes of cell migration and proliferation—the key characteristics of which determine cancer aggression. On the one hand, cells undergoing malignization utilize the mechanisms involved in embryogenesis and ontogenesis in general, and on the other hand, disturbances and defects in these mechanisms restrict their regulation by the host organism. However, cells, while going through their life cycles, having their structures altered, and suffering genetic mutations, are a subject to natural selection within the body. They may become potentially vulnerable to changes in their microenvironment and can adapt their metabolism accordingly. Therefore, the cancer cells initially generate a more rigid matrix than such presented in healthy tissues, not because it is beneficial for them, but because they are under the influence of nonspecific natural selection, which secures favorable variations of gene expression profiles suitable for the life under certain conditions. Thus, a possible treatment strategy would be to inhibit the expression of certain proteins of the extracellular matrix, and then alter its structure to enhance the cytostatic properties of treatment drugs.

## Supplementary Materials

The following are available online at https://www.mdpi.com/article/10.3390/biomedicines10020345/s1, Table S1: Types of cytoskeletal proteins, its distribution in human organism according to the Human protein atlas and J. Schweizer et al. (2006) [167].
