# Supplementary material for: Cell and Tissue Nanomechanics: From Early Development to Carcinogenesis
Source: Biomedicines. 2022 Feb 1;10(2):345. doi: 10.3390/biomedicines10020345 (PMC8961777; doi:10.3390/biomedicines10020345)
Supplement: Supplementary file 1 [file biomedicines-10-00345-s001.zip › biomedicines-1518391-supplementary.pdf]

biomedicines

Table S1: Types of cytoskeletal proteins, its distribution in human organism according to the Human protein atlas and J. Schweizer et. al (2006) [139].

Protein type	Protein	Gene name	Tissue distribution	Tumor prognostic marker
IF Type 1	Keratin 9	KRT9	Sole of foot, Adrenal gland	Not prognostic, enriched in melanoma
	Keratin 10	KRT10	skin	Unfavorable in renal cancer, enriched in melanoma
	Keratin 12	KRT12	Cornea	Not prognostic, detected in some tumors
	Keratin 13	KRT13	Oral mucosa, esophagus, urinary bladder, vagina, cervix, uterine	Unfavorable in renal cancer, pancreatic cancer; detected in many tumors
	Keratin 14	KRT14	Oral mucosa, esophagus, prostate, vagina, cervix, uterine, breast	Favorable in breast cancer; group enriched (cervical cancer, head and neck cancer, melanoma); detected in many tumors
	Keratin 15	KRT15	Bronchus, oral mucosa, salivary gland, esophagus, duodenum, small intestine, colon, rectum, prostate, vagina, breast, heart muscle, skin, hair, appendix, tonsil	Favorable in breast cancer; enriched in cervical cancer
	Keratin 16	KRT16	Lung, oral mucosa, skin, tonsil	Unfavorable in pancreatic cancer; group enriched (cervical cancer, head and neck cancer, melanoma); detected in many tumors.
	Keratin 17	KRT17	Nasopharynx, bronchus, oral mucosa, salivary gland, esophagus, pancreas, urinary bladder, testis, epididymis, seminal vesicle, prostate, fallopian tube, breast, tonsil	Unfavorable in renal cancer, favorable in breast cancer; cancer enhanced (head and neck cancer); detected in all tumors.

Keratin 18	KRT18	Thyroid gland, parathyroid gland, adrenal gland, nasopharynx, bronchus, lung, salivary gland, stomach, duodenum, small intestine, colon, rectum, liver, gallbladder, pancreas, kidney, urinary bladder, epididymis, seminal vesicle, prostate, fallopian tube, endometrium, cervix, uterine, placenta, breast, appendix	Unfavorable in pancreatic cancer and in lung cancer; low cancer specificity; detected in all tumors.
Keratin 19	KRT19	Thyroid gland, parathyroid gland, nasopharynx, bronchus, lung, oral mucosa, salivary gland, esophagus, stomach, duodenum, small intestine, colon, rectum, liver, gallbladder, pancreas, kidney, urinary bladder, epididymis, seminal vesicle, prostate, vagina, fallopian tube, endometrium, cervix, uterine, placenta, breast, skin, appendix, tonsil	Unfavorable in renal and in pancreatic cancer; low cancer specificity; detected in many tumors.
Keratin 20	KRT20	Stomach, duodenum, small intestine, colon, rectum, urinary bladder, appendix	Unfavorable in renal and in liver cancer; group enriched (colorectal cancer, stomach cancer, urothelial cancer; detected in many tumors.

Keratin 23	KRT23	Cerebral cortex, thyroid gland, parathyroid gland, adrenal gland, respiratory system, digestive system (exclude liver), genitourinary system, muscles, soft tissue, skin, appendix, tonsil, tonsil, bone marrow.	Unfavorable in urothelial cancer; low cancer specificity; detected in many tumors.
Keratin 24	KRT24	Detected in all organs exclude cerebellum, hippocampus, caudate, spleen, lymph node	Not prognostic, cancer enhanced (head and neck cancer); detected in some tumors.
Keratin 25	KRT25	Hair	Not prognostic, not detected in cancer
Keratin 26	KRT26	Hair	Not prognostic, not detected in cancer
Keratin 27	KRT27	Hair	Not prognostic, cancer enhanced (endometrial cancer); detected in some tumors.
Keratin 28	KRT28	Hair	Not prognostic, cancer enhanced (cervical cancer, endometrial cancer); detected in some tumors.
Keratin 31	KRT31	Cerebral cortex, cerebellum, olfactory region, pons and medulla, corpus callosum, spinal cord, salivary gland, esophagus, cervix, uterine, breast, skeletal muscle, adipose tissue, skin, thymus, tonsil	Not prognostic, low cancer specificity, detected in many tumors.
Keratin 32	KRT32	Pending normal tissue annotation.	Not prognostic, not detected in cancer
Keratin 33A	KRT33A	Hair	Not prognostic, cancer enhanced (urothelial cancer); detected in some tumors.

	Keratin 33B	KRT33B	Hair	Not prognostic, cancer enhanced (urothelial cancer); detected in single tumors.
	Keratin 34	KRT34	Hair	Not prognostic, cancer enhanced (head and neck cancer); detected in some tumors.
	Keratin 35	KRT35	Hair	Not prognostic, not detected in cancer
	Keratin 36	KRT36	Pending normal tissue annotation.	Not prognostic, cancer enhanced (head and neck cancer); detected in single tumors.
	Keratin 37	KRT37	Pending normal tissue annotation.	Not prognostic, not detected in cancer
	Keratin 38	KRT38	Pending normal tissue annotation.	Not prognostic, group enriched (head and neck cancer, urothelial cancer); detected in some tumors.
	Keratin 39	KRT39	Pending normal tissue annotation.	Not prognostic, not detected in cancer
	Keratin 40	KRT40	Adrenal gland, stomach, colon, testis, soft tissue, hair.	Not prognostic, cancer enhanced (colorectal cancer); detected in some tumors.
IF Type 2	Keratin 1	KRT1	Oral mucosa, esophagus, vagina, cervix, uterine, skin	Not prognostic, group enriched (head and neck cancer, melanoma); detected in many tumors.
	Keratin 2	KRT2	Esophagus, vagina, cervix, utterine, skin.	Not prognostic, group enriched (head and neck cancer, melanoma); detected in some tumors.
	Keratin 3	KRT3	Pending normal tissue annotation.	Not prognostic, cancer enhanced (head and neck cancer); detected in some tumors.

Keratin 4	KRT4	Nasopharynx, bronchus, oral mucosa, esophagus, vagina, cervix, uterine, tonsil	Unfavorable in ovarian and urothelial cancer; group enriched (cervical cancer, head and neck cancer, stomach cancer, urothelial cancer); detected in many cancer.
Keratin 5	KRT5	Nasopharynx, bronchus, oral mucosa, salivary gland, esophagus, urinary bladder, epididymis, seminal vesicle, prostata, vagina, cervix, uterine, breast, skin, tonsil	Favorable in breast cancer, cancer enhanced (head and neck cancer), detected in many tumors.
Keratin 6A	KRT6A	Nasopharynx, bronchus, oral mucosa, esophagus, vagina, cervix, uterine, skin, tonsil	Unfavorable in lung cancer; cancer enhanced (head and neck cancer), detected in many tumors.
Keratin 6B	KRT6B	Nasopharynx, bronchus, oral mucosa, esophagus, vagina, cervix, uterine, skin, tonsil	Favorable in breast cancer, unfavorable in urothelial cancer; cancer enhanced (head and neck cancer), detected in many tumors.
Keratin 6C	KRT6C	Nasopharynx, bronchus, oral mucosa, esophagus, vagina, cervix, uterine, skin, tonsil	Unfavorable in lung cancer; cancer enhanced (head and neck cancer), detected in many tumors.
Keratin 7	KRT7	Thyroid gland, parathyroid gland, nasopharynx, bronchus, lung, salivary gland, esophagus, stomach, liver, gallbladder, pancreas, kidney, urinary bladder, epididymis, seminal vesicle, prostate, fallopian tube, endometrium, cervix, uterine, placenta, breast	Favorable in renal cancer, unfavorable in pancreatic, ovarian and in endometrial cancer; cancer enhanced (urothelial cancer); detected in many tumors.

Keratin 8	KRT8	Thyroid gland, parathyroid gland, nasopharynx, bronchus, lung, salivary gland, stomach, duodenum, small intestine, colon, rectum, liver, gallbladder, pancreas, kidney, urinary bladder, testis, epididymis, seminal vesicle, prostate, ovary, fallopian tube, endometrium, cervix, uterine, placenta, breast, appendix, tonsil	Unfavorable in pancreatic cancer; low cancer specificity, detected in many tumors.
Keratin 71	KRT71	Hair	Not prognostic, cancer enhanced (endometrial cancer); detected in single tumors.
Keratin 72	KRT72	Hair	Not prognostic; not detected in tumors.
Keratin 73	KRT73	Hair	Not prognostic; not detected in tumors.
Keratin 74	KRT74	Hair	Not prognostic; not detected in tumors.
Keratin 75	KRT75	Nasopharynx, bronchus, oral mucosa, salivary gland, esophagus, colon, rectum, urinary bladder, epididymis, seminal vesicle, prostate, vagina, cervix, uterine, skin, hair, tonsil	Not prognostic, cancer enhanced (head and neck cancer); detected in many tumors.
Keratin 76	KRT76	Oral mucosa, vagina, skin, tonsil	Not prognostic, cancer enhanced (head and neck cancer); detected in single tumors.
Keratin 77	KRT77	Hippocampus, oral mucosa, vagina, cervix uterine, skin	Not prognostic; low cancer specificity; detected in some tumors.

Keratin 78	KRT78	Oral mucosa, esophagus, vagina, cervix, uterine, skin, tonsil	Not prognostic, cancer enhanced (head and neck cancer); detected in many tumors.
Keratin 79	KRT79	Nasopharynx, bronchus, oral mucosa, salivary gland, esophagus, vagina, cervix, uterine, breast, skin, hair, tonsil	Not prognostic; group enriched (breast cancer, cervical cancer, head and neck cancer, melanoma, urothelial cancer); detected in some tumors
Keratin 80	KRT80	Thyroid gland, parathyroid gland, adrenal gland, lung, salivary gland, esophagus, tongue, colon, rectum, gallbladder, pancreas, kidney, urinary bladder, testis, epididymis, seminal vesicle, prostate, ductus deferens, vagina, ovary, fallopian tube, endometrium, cervix, uterine, placenta, breast, skin, tonsil	Unfavorable in breast cancer, low cancer specificity, detected in many tumors.
Keratin 81	KRT81	Hair	Not prognostic, cancer enhanced (breast cancer, cervical cancer); detected in many tumors.
Keratin 82	KRT82	Hair	Not prognostic; not detected in tumors.
Keratin 83	KRT83	Hair	Not prognostic, cancer enhanced (thyroid cancer); detected in some tumors.
Keratin 84	KRT84	Adipose tissue, hair	Not prognostic, cancer enhanced (head and neck cancer); detected in single tumors.
Keratin 85	KRT85	Hair	Not prognostic; not detected in tumors.

	Keratin 86	KRT86	Hair	Not prognostic; low cancer specificity, detected in many tumors.
IF Type III	Vimentin	VIM	Cerebral cortex, hippocampus, caudate, thyroid gland, adrenal gland, nasopharynx, bronchus, lung, colon, liver, pancreas, kidney, testis, epididymis, seminal vesicle, vagina, ovary, fallopian tube, endometrium, cervix, uterine, placenta, breast, soft tissue, adipose tissue, skin, appendix, splen, lymph node, tonsil, bone marrow	Favorable in endometrial cancer, unfavorable in renal cancer; low cancer specificity, detected in all tumors.
	Desmin	DES	Heart muscle, smooth muscle, skeletal muscle	Unfavorable in renal cancer; cancer enhanced (prostate cancer); detected in all tumors.
	Glial fibrilar acidic protein (GFAP)	GFAP	Cerebral cortex, cerebellum, hippocampus, caudate, testis	Not prognostic; cancer enriched (glioma), detected in some tumors.
	Peripherin	PRPH	Dorsal raphe, adrenal gland, colon, testis, soft tissue	Unfavorable in glioma; low cancer specificity; detected in some cancer.
IF Type IV	Neurofilament light NF-L	NEFL	Cerebral cortex, cerebellum, hippocampus, soft tissue	Unfavorable in renal cancer; cancer enhanced (renal cancer); detected in many tumors.
	Neurofilament medium NF-M	NEFM	Cerebral cortex, cerebellum, hippocampus, caudate, colon, placenta, soft tissue	Not prognostic; low cancer specificity, detected in many tumors.
	Neurofilament heavy NF-H	NEFH	Cerebral cortex, cerebellum, hippocampus, caudate, colon, prostate, ovary, soft tissue	Favorable in breast cancer; group enriched (prostate cancer); detected in many tumors.

	α-Internexin	INA	Cerebral cortex, cerebellum, hippocampus, caudate, adrenal gland, testis	Not prognostic; cancer enhanced (testis cancer); detected in many tumors.
IF Type v	Lamin A<br>Lamin C	LMNA	Express in all organs	Not prognostic; low cancer specificity; detected in all tumors.
	Lamin B1	LMNB1	Express in all organs	Unfavorable in renal cancer and in liver cancer; low cancer specificity; detected in all tumors.
	Lamin B2	LMNB2	Express in all organs	Favorable in stomach cancer, unfavorable in melanoma, renal cancer, liver cancer and in prostate cancer; low cancer specificity; detected in all tumors.
IF Type VI	Nestin	NES	Cerebral cortex, cerebellum, colon, kidney, testis, breast, heart muscle, smooth muscle, skeletal muscle, soft tissue	Unfavorable in renal cancer; group enriched (glioma, melanoma, testis cancer); detected in all tumors.
Actin proteins	Actin beta	ACTB	Cerebral cortex, lung, salivary gland, stomach, duodenum, small intestine, colon, rectum, gallbladder, pancreas, kidney, testis, epididymis, endometrium, placenta, breast, heart muscle, smooth muscle, skeletal muscle, adipose tissue, appendix, splen, lymph node, tonsil, bone marrow	Unfavorable in renal cancer and in head and neck cancer; low cancer specificity; detected in all tumors.
	Actin gamma 1	ACTG1	Express in all organs	Favorable in colorectal cancer; low cancer specificity; detected in all tumors.

	Actin alpha 1 skeletal muscle	ACTA1	Breast, all types of muscles	Not prognostic; group enriched (head and neck cancer, prostate cancer); detected in many tumors.
	Actin alpha 2 smooth muscle, aorta	ACTA2	Kidney, ovary, endometrium, breast, smooth muscle	Unfavorable in renal cancer; low cancer specificity; detected in all tumors.
	Actin alpha cardiac muscle 1	ACTC1	Placenta, all types of muscles	Unfavorable in head and neck cancer, urothelial cancer; group enriched (head and neck cancer, prostate cancer, testis cancer, urothelial cancer); detected in many tumors.
	Actin gamma 2 smooth muscle enteric	ACTG2	lung, stomach, duodenum, small intestine, colon, rectum, liver, gallbladder, pancreas, kidney, testis, epididymis, breast, smooth muscle, appendix, lymph node, tonsil	Unfavorable in renal cancer, cancer enhanced (prostate cancer, stomach cancer); detected in all tumors.
Microtubules	Tubulin alpha 1a	TUBA1A	Express in all organs	Unfavorable in renal cancer; cancer enriched (glioma); detected in all tumors.
	Tubulin alpha 1b	TUBA1B	Express in all organs exclude skeletal muscle.	Unfavorable in renal cancer, liver cancer, breast cancer; low cancer specificity; detected in all tumors.
	Tubulin alpha 1c	TUBA1C	Express in all organs exclude skeletal muscle.	Favorable in colorectal cancer, unfavorable in liver cancer, renal cancer, pancreatic cancer, breast cancer, lung cancer; low cancer specificity; detected in all tumors.
	Tubulin alpha 3c	TUBA3C	Testis	Not prognostic; low cancer specificity; detected in many tumors.

Tubulin alpha 3d	TUBA3D	RNA-based expert annotation could not be performed, due to inconclusive results.	Unfavorable in renal cancer, favorable in breast cancer; low cancer specificity; detected in many tumors.
Tubulin alpha 3e	TUBA3E	RNA-based expert annotation could not be performed, due to inconclusive results.	Not prognostic; low cancer specificity; detected in some tumors.
Tubulin alpha 4a	TUBA4A	Express in all organs exclude skeletal muscle.	Unfavorable in liver cancer; low cancer specificity; detected in all tumors
Tubulin alpha 4b	TUBA4B	Pending normal tissue annotation.	Not prognostic; cancer enriched (endometrial cancer); detected in some tumors.
Tubulin alpha 8	TUBA8	Express in all organs exclude skeletal muscle	Not prognostic, not detected in cancer.
Tubulin beta class I	TUBB	Express in all organs exclude cerebellum, parathyroid gland, placenta, muscles.	Unfavorable in renal cancer and in liver cancer; low cancer specificity; detected in all tumors.
Tubulin beta 2A class IIa	TUBB2A	Cerebral cortex, hippocampus, nasopharynx, bronchus, oral mucosa, stomach, duodenum, small intestine, colon, rectum, testis, epididymis, fallopian tube, breast, skin	Unfavorable in urothelial cancer, favorable in renal cancer; low cancer specificity; detected in all tumors.
Tubulin beta 2B class IIb	TUBB2B	Cerebral cortex, hippocampus, nasopharynx, bronchus, oral mucosa, stomach, duodenum, colon, rectum, testis, epididymis, fallopian tube, breast, skin	Unfavorable in endometrial cancer; cancer enriched (glioma); detected in many tumors.

Tubulin beta 3 class III	TUBB3	Cerebral cortex, hippocampus, caudate, nasopharynx, bronchus, oral mucosa, stomach, duodenum, colon, rectum, testis, epididymis, fallopian tube, breast, soft tissue, skin	Not prognostic; low cancer specificity; detected in many tumors.
Tubulin beta 4A class IVa	TUBB4A	Cerebral cortex, hippocampus, adrenal gland, nasopharynx, bronchus, oral mucosa, stomach, duodenum, colon, rectum, testis, epididymis, fallopian tube, breast, skin	Unfavorable in endometrial cancer; group enriched (glioma, melanoma, testis cancer); detected in many tumors.
Tubulin beta 4B class IVb	TUBB4B	Express in all organs exclude cerebellum, parathyroid gland, placenta, muscles.	Favorable in thyroid cancer, endometrial cancer, unfavorable in liver cancer; low cancer specificity, detected in all tumors.
Tubulin beta 6 class V	TUBB6	Express in all tissues exclude cerebellum, parathyroid gland, placenta, muscles.	Unfavorable in renal cancer and in urothelial cancer; low cancer specificity; detected in all tumors.
Tubulin beta 1 class VI	TUBB1	Express in all organs exclude cerebellum, parathyroid gland, muscles.	Not prognostic, not detected in cancer.
Tubulin beta 8 class VIII	TUBB8	RNA-based expert annotation could not be performed, due to inconclusive results.	Not prognostic; cancer enriched (testis cancer); detected in single tumors.
Tubulin gamma 1	TUBG1	Express in all organs exclude hippocampus, esophagus, liver, heart and skeletal muscles.	Unfavorable in liver cancer, renal cancer, favorable in endometrial cancer; low cancer specificity; detected in all tumors.
Tubulin gamma 2	TUBG2	Express in all organs.	Unfavorable in liver cancer and in renal cancer; low cancer specificity; detected in all tumors.

Tubulin delta 1	TUBD1	Express in all organs.	Unfavorable in liver cancer and in renal cancer, favorable in colorectal cancer; low cancer specificity; detected in all tumors.
Tubulin epsilon 1	TUBE1	Express in all organs exclude ovary and smooth muscle.	Not prognostic; low cancer specificity; detected in all tumors.
